# Mothers’ experiences of reducing family mealtime screen use in Australian households with young children

**DOI:** 10.1017/S1368980023002847

**Published:** 2023-12-15

**Authors:** Eloise-Kate Litterbach, Rachel Laws, Miaobing Zheng, Karen J Campbell, Alison C Spence

**Affiliations:** 1 Institute for Physical Activity and Nutrition (IPAN), School of Exercise and Nutrition Sciences, Deakin University, Burwood, Australia; 2 The Australian Centre for Behavioural Research in Diabetes, Diabetes Victoria, Melbourne, VIC 3053, Australia

**Keywords:** Family meals, Screen use, Television, Handheld device, Young children, Motivational interviewing, Behaviour change

## Abstract

**Objective:**

Screen use at mealtimes is associated with poor dietary and psychosocial outcomes in children and is disproportionately prevalent among families of low socio-economic position (SEP). This study aimed to explore experiences of reducing mealtime screen use in mothers of low SEP with young children.

**Design:**

Motivational interviews, conducted via Zoom or telephone, addressed barriers and facilitators to reducing mealtime screen use. Following motivational interviews, participants co-designed mealtime screen use reduction strategies and trialled these for 3–4 weeks. Follow-up semi-structured interviews then explored maternal experiences of implementing strategies, including successes and difficulties. Transcripts were analysed thematically.

**Setting:**

Australia.

**Participants:**

Fourteen mothers who had no university education and a child between six months and six years old.

**Results:**

A range of strategies aimed to reduce mealtime screen use were co-designed. The most widely used strategies included changing mealtime location and parental modelling of expected behaviours. Experiences were influenced by mothers’ levels of parenting self-efficacy and mealtime consistency, included changes to mealtime foods and an increased value of mealtimes. Experiences were reportedly easier, more beneficial and offered more opportunities for family communication, than anticipated. Change required considerable effort. However, effort decreased with consistency.

**Conclusions:**

The diverse strategies co-designed by mothers highlight the importance of understanding *why* families engage in mealtime screen use and providing tailored advice for reduction. Although promising themes were identified, in this motivated sample, changing established mealtime screen use habits still required substantial effort. Embedding screen-free mealtime messaging into nutrition promotion from the inception of eating will be important.

The early years are a crucial time for developing healthy eating behaviours, shown to track from infancy into adulthood^(1,2)^. Currently, many children’s diets are far from optimal. From as early as around 3·5 years, intakes of fruit and vegetables do not meet national dietary guidelines for optimal health^(3–5)^. In addition, > 90 % of Australian children from the age of 9 months exceed the upper recommended limit of discretionary foods (energy-dense, nutrient poor foods such as cakes, biscuits, chips, ice cream and sugar sweetened beverages)^(3,4)^. This is mirrored in other high-income countries, such as the USA^(6)^. Furthermore, poor dietary outcomes are more common in children from families of low socio-economic position (SEP)^(7)^, even before children reach one year old^(3)^.

A useful setting to promote healthy eating to children, regardless of SEP, is through the home environment, given the strongest correlates of child diet quality are home based^(8)^. Before children reach primary school age (around 6 years in Australia), most of a child’s food intake occurs within the home, even for children who attend long day care^(9)^. In addition, recent evidence suggests that meals at home tend to include fewer fruits and vegetables than those provided at childcare^(10)^, highlighting the vast potential to improve child diets within the home setting. Family meals, where a child and caregiver eat together, are part of this home environment. Frequently engaging in family mealtimes is cross sectionally associated with optimal nutritional and developmental outcomes for children^(11)^, allowing family members to come together for regular communication and parental modelling of expected behaviours. Family mealtimes present opportunities for offering and consuming healthy foods and in turn, for children to develop healthy food related behaviours. In Australia, 77 % of families with young children engage in family mealtimes most nights^(12)^, and families are interested in and motivated by the prospect of family meals^(13)^ making it an ideal context in which to promote healthy eating behaviours.

More than one-third of young children in Australia^(12)^, the USA^(14)^ and the United Kingdom^(15)^ engage in screen use (television, tablets and smartphones) during mealtimes, every day. Additionally, children from families of lower SEP may be most likely to engage in mealtime screen use^(16)^. Evidence suggests that mealtime screen use reduces opportunities for family cohesion at mealtimes^(17)^, and cross-sectional^(11)^ and 2-year prospective evidence^(18)^ suggests that children’s diets are poorer when screens are included in family meals. Qualitative research with diverse^(19)^ and low-income^(20)^ families has also found that many parents acknowledge mealtime screen use as not aligning with best practice eating environments and that turning the TV off would improve family communication and connection. However, this awareness does not appear to be enough to guide behaviour.

A useful framework to understand and inform behaviour change is the Capability, Opportunity, Motivation and Behaviour (COM-B) System of Behaviour Change^(21)^. This theory posits that behaviour occurs through an interacting system whereby one’s capability (having the skills and knowledge to enact a behaviour) and opportunity (having access to information or equipment and relevant social and cultural norms) influence one’s motivation to enact a behaviour (thoughts and feelings towards engaging in the behaviour, such as goals, habits and emotions). The behaviour itself also has a unidirectional relationship with each of capability, opportunity and motivation^(21)^.

Understanding caregivers’ capability, opportunity and motivation around mealtime screen use is important in establishing whether reducing screens at mealtimes is feasible for families, especially for families of low SEP who may experience an increased number of barriers to healthy mealtime behaviours^(20,22)^.

Recent evidence from families of low SEP suggests that families engage in mealtime screen use to manage the difficulty of making family meals happen, to reduce picky eating^(20)^ and to potentially manage children’s negative emotions^(23)^. Screen use immediately prior to mealtimes has been reported to extend into the mealtime^(22)^. Large-scale interventions have employed reducing mealtime screen use as a strategy for promoting nutrition through the family meals setting in older children^(24)^ and young children^(25)^. However, no studies have explored caregiver experiences of reducing mealtime screen use in families with young children, making it difficult to determine whether limiting mealtime screen use is a feasible goal for families.

Receptiveness to interventions can be enhanced by tailoring strategies to address barriers^(26)^ and aligning these with what is already understood through strength-based approaches^(27)^. However, strategies based on researchers’ interpretations of qualitative inquiry are not always understood and translated as the participant intended. Therefore, the co-construction of ideas between researchers and participants, based upon participants’ lived experiences and researchers’ scientific knowledge^(28)^, is a useful way to design behaviour change strategies. A co-design approach involves conducting research *with* participants, rather than *on* them, may increase acceptance and engagement^(26,28)^ and therefore has the potential to be more respectful and effective. Planning research collaboratively with members of the community impacted by the implementation of research outcomes also integrates end-user value and the planning of broader dissemination at the core of the research^(29)^.

Utilising the COM-B System of Behaviour Change as a framework allows for a greater understanding of how behaviour change strategies can be tailored to increase caregivers’ capability, opportunity and motivation for change and helps to identify underlying ideas, broader meanings and implications that inform behaviour change.

Therefore, this study aimed to co-design strategies tailored to the challenges of mealtimes and explore caregiver experiences of using these strategies to reduce mealtime screen use, in families of low SEP with young children.

## Methods

This study reports on the second part of a two-part project. Part one was an exploratory study of mealtime screen use behaviours in 25 mothers with young children^(30)^. Part two, the focus of this study, involved a sub-group of participants ‘opting in’ to a motivational interviewing (MI) and strategy co-design session, followed by a brief trial period, and then a follow-up interview for each participant.

### Research paradigm and reflexivity

This research project was underpinned by Constructionism. This theory is driven by a socially constructed idea of continually changing reality and the belief that our understanding of knowledge within reality is indeterminate^(31)^. Within Constructionism, this research used a combination of Constructivist and Critical Theory paradigms. Constructivism offers that as humans, we create truth and reality individually and together^(32)^, supporting the use of in-depth interviews for the initial stages of this research. In addition, elements of Critical Theory were included, whereby researchers act as advocates for the participant and challenge dominant discourse and data collection methods which previously reflect the researcher as the only expert^(33)^. This combined paradigm approach aimed to promote a respectful interchange between researcher and participant. Facilitating a sense of ownership of the research process and voice in the scientific literature for participants was important^(33)^ whilst acknowledging that the presented interpretations were a co-creation between researcher and participants.

It is also important to acknowledge the researchers’ backgrounds given they impact the research. All researchers are qualified nutritionists and mothers. The lead researcher also having a background in Psychology, Healthy Conversation Skills^(34)^ and extensive experience in qualitative interviewing.

### Eligibility, reimbursement, consent and ethics approval

Participants were eligible if they lived in Australia, could speak and read English, were a parent or caregiver of at least one child between six months and six years of age and did not have a university degree. Criterion regarding education level was informed by previous research, which describes parental education as a proxy for family SEP^(3)^. Additionally, maternal education is reported to be a good predictor of children’s diet and screen behaviours^(35)^. A supermarket voucher ($40AUD) was provided on completion of part one, and then again for those participating in part two. Participants provided informed consent both online (recruitment) and verbally (each interview). Ethics for this study was approved by Deakin University (HEAG-H 178_2020).

### Recruitment

Recruitment via social media was conducted from January to July 2021, when many Australian cities and suburbs were experiencing strict COVID-19 related lockdowns. Facebook™ advertising attracted interested caregivers to a brief online eligibility, consent and registration form using REDCap™ (V10·6·0). Participants were then contacted via email or phone to arrange a convenient time for a 45-minute interview with the researcher.

#### Initial interview (part one)

Twenty-five mothers completed an initial interview aimed to explore why families may use screens at mealtimes (part one, published elsewhere)^(30)^. At commencement, participants were informed that part way through the interview the researcher may ask the participant of their interest in participating in further related research.

#### Motivational interviewing and co-design (part two)

Fifteen parents were invited to continue to part two, comprising those families who engaged in any mealtime screen use and discussed an openness to change. Part two offered the opportunity to participate in an individual MI session, and to co-design strategies for reducing mealtime screen use to apply in their home. Participants were provided the option of completing the MI session immediately or, to go away and consider their interest in participating, and later scheduling a time to complete the individual MI session. Fourteen of the 15 invited mothers agreed to participate in this step, formed the sub-sample for this study and all participated in MI sessions immediately. Participants were asked to trial their co-designed strategies for three to four weeks, then recontacted and invited to discuss experiences via Zoom or telephone interview.

### Data collection

Data collection focused on a co-construction of ideas and understanding between the researcher and participant^(31)^. Researchers acknowledge the elements of Critical Theory embedded into the methods utilised, whereby researchers worked collaboratively with participants to identify and implement their own ideas, supported by the researcher.

#### Motivational interviewing

All MI sessions were conducted by the lead author. After initial exploration of current behaviours (part one), participants spent 20–30 min with the researcher in exploring desired outcomes and anticipated difficulties, goal setting and planning 2–4 tailored strategies aimed to reduce mealtime screen use. This process aimed to develop behaviour change strategies through collaboration and reflection of participants’ lived experiences and researchers’ scientific knowledge. Goals were participant-led and could include the reduction of any type of screen (TV, tablet or phone use), at any meal or snack time and for any number of mealtimes across the week. A semi-structured interview script, provided in Additional file 1, was used to help guide the conversation towards study aims and to ensure goal setting was comprehensive. A SMARTER ME (SMARTER: Specific, Measurable, Achievable, Realistic, Time based, Evaluate, Reflect, ME: Motivation, End goal) goal setting plan was used as a guide^(34)^. MI is driven by a conversational style of interviewing, which helps participants explore their own motivations around a particular behaviour and commitment to changing it^(36)^. A key aspect of MI is helping people to weigh up costs and benefits to help them move towards such change^(37)^. Participants were encouraged to identify factors influencing their family’s mealtime screen use and create a comprehensive plan. The MI and co-design session provided *Problem Solving* (BCT #1·2), *Emotional Social Support* (BCT #3·3) and *Practical Social Support* (BCT #3·2), as well as facilitating the development of further behaviour change techniques (BCT) according to Michie and colleagues’ BCT taxonomy^(38)^. Mothers came up with between two and four specific strategies for their family to trial, which were summarised and emailed back to each participant following the MI and co-design session.

#### Trial phase and follow-up interviews

Participants were encouraged to document thoughts, feelings, ideas or changes to the planned strategies during the trial period and were offered the opportunity to contact the researcher for support during this time.

Around 3–4 weeks after the initial interview and MI, to offer enough opportunity for trialing strategies, participants were invited to complete a follow-up interview. Using the COM-B System of Behaviour Change as a framework, a semi-structured interview script allowed the researcher to map if and how the elements of COM-B were interacting to influence mealtime screen use and behaviour change^(21)^. Additional file 2 outlines the follow-up interview questions, mapped to COM-B.

All interviews were recorded with permission and professionally transcribed using Digital Transcripts©. Transcripts were checked by the lead author for accuracy, annotated to include visual cues or gestures made in the video and de-identified for storage.

### Analysis

Analyses were conducted by the lead author, who was also the interviewer, strengthening the credibility of the coding process alongside achieving continuity of interview techniques, persistent observation and prolonged engagement with the data^(39)^. Analysis for this study involved an iterative process which began immediately after each interview whereby the researcher took notes about observations and musings regarding the exchange. In addition, so the researcher could frequently reflect on the research development, a continually iterated running sheet of overall thoughts pertaining to interviews, possible correlations, ideas to explore and potential questions for prospective interviews was kept. This was referred to continuously during the analysis phase.

Upon the completion of data collection phase, the transcripts, annotations and other documents about the interviews were re-read for familiarity and the separate interactions for each participant were merged into one document. Semantic level reflexive thematic analysis^(40)^ was conducted, using NVivo. Semantic level thematic analysis focuses on demonstrating patterns in surface level content (without trying to identify latent meanings), interpreting those patterns and highlighting the implications and broader meanings^(41)^. To begin, the lead author inductively created codes, which grouped similar ideas within the data. Once all interviews were coded, patterns within the codes were identified and tentatively labelled. All five authors were involved in an iterative process, where codes were discussed, merged, became sub-codes and or were renamed, until the coding framework reflected the data and aligned with the aims of the study. Behaviour change strategies were identified, grouped by likeness and labelled intuitively. Finally, BCT were mapped to components of COM-B, condensed accordingly and classified using Michie and colleagues’ BCT taxonomy^(38)^.

## Results

### Participants

Fourteen mothers participated in the MI session, trial period and follow-up interview (aged 29–44 years). Table 1 illustrates participant demographic characteristics. Most had completed a certificate or diploma, and ten mothers had two or more children. Nine lived in a dual-headed household, three were born in a country other than Australia and one mother was of Aboriginal descent. Most (twelve) families engaged in family meals > 5 times per week, with reported mealtime screen use varying from one meal per day to every meal (data not shown). The types of screens engaged at mealtimes varied from meal to meal and included television use, child handheld device use (individual and shared) and caregiver phone use, depending on the needs and desires of family members.

**Table 1:** Participant demographic characteristics

Demographic characteristics *n* 14	
Highest level of completed education	
Certificate/diploma (e.g. childcare, technician)	10
Trade/apprenticeship (e.g. hairdresser, chef)	2
Primary or high school education	2
Country of birth	
Australia	11
Bangladesh	1
India	1
United Kingdom	1
Dual headed household (yes)	9
Number of children per family (age range 7 months–16 years^*^)	
One	4
Two	6
Three	2
Four	2

* All families had ≥ one child between the age of 6 months and 6 years.

### Motivational interviewing: *Mothers’ considerations when planning to make change*

During the MI phase, mothers discussed perceived ideal environments for change and acknowledged that change would be most difficult outside of these environments. For example, it would be more difficult to limit mealtime screen use when their partner was not around for support, or if their child(ren) were tired or grumpy, with some reporting an anticipated lack of confidence to engage in strategies in these situations. Most mothers acknowledged the need to be prepared for their child to resist change while establishing a new routine. Table 2 shows mothers’ anticipated challenges and desired outcomes of reducing mealtime screen use, along with example quotes. In summary, while mothers anticipated a range of challenges, their desire for outcomes around connection and engagement, exposure to healthy environments and family connectedness, helped to limit these anticipated challenges. For example, some mothers were worried about how they would manage their child’s reactions to screen-free meals. These mothers expressed concern that if their child reacted negatively, mothers might feel upset or grumpy. However, mothers reported that they were motivated to stay calm and avoid yelling or getting upset at their child in order to help promote a calm and positive atmosphere.

**Table 2:** Mothers’ anticipated challenges and desired outcomes of reducing mealtime screen use

Anticipated challenges	Example quote
Breaking old habits Young child (< 2 years) Older child (> 4 years)	‘It’s just getting out of the habit, I think. I don’t think I’ll have much resistance. He’s not like totally obsessed, you know, with having the TV on all the time.’ ‘I think it’s gonna be hard because the kids get really addicted especially my son, he’s really addicted to screens.’
Household layout/resources TV is visible from eating space No dedicated eating space	‘…the environment also…my dining space is not in separate room. So, they can easily watch TV from this side, they can easily ask, I want to watch TV.’ ‘We do (have a dining table)…it’s kind of got computers on it at the moment. It exists…more likely scenario is if we made like maybe like a picnic or something like that instead.’
Managing child behaviour Children wandering from table Siblings arguing Children protesting/crying Challenges of more than one child	‘…they’d be wandering, they’d be running away…especially the older one…we have to scream two, three times to come back. Some days I have to like literally stand next to him holding his hand.’ ‘Trying to get the older two not to fight.’ ‘He’s just gonna scream and scream and say, I want the TV.’ ‘It’s the only way that we can make sure she’s gonna stay in one spot, if we have to go and do something with the baby.’
Parent response to child behaviour Frustrated/ Snappy/ Cannot be bothered	‘Potential for more frustration or more moodiness from me.’ ‘…those difficult times where I just go, ‘oh I’m so tired I just can’t be bothered with it all’.’

Desired outcomes	Example quote
More family connection	‘I just would like to be more connected like, particularly with my daughter…there’s a lot of missed opportunities to relationship build with her, during those [meal] times.’
Child more present/aware	‘I think he would be more engaged in what’s around him.’
Less guilt	‘I’d feel less guilty. That’d be good.’
Child experience healthy role modelling	‘I mean it’s mainly I would love to see like, if maybe this [child], if she was watching everyone eating as well…Maybe it would be more enjoyable for her, if she was like sitting around, watching her siblings and talking to them, joining in that way instead?’
Child will sit still/quietly	‘If my kids sit quietly, if the older one doesn’t run away if he eats quietly then obviously, I don’t need to switch on the screen.’

### Motivational interviewing: *Co-designed strategies (planned and engaged)*

Most strategies were generated from mothers’ reflections around *why* they used screens at mealtimes, as well as past experiences, considering things that had or had not worked before^(30)^. Mothers anticipated the ways their child might respond to change and were receptive to the differing needs of their children of varying ages. These were carefully considered in planning, and strategies were tailored accordingly. Participants co-designed a wide variety of strategies, identified in Table 3. Strategies were also mapped to corresponding BCT, which encompassed broad BCT groups; 1. Goals and Planning, 3. Social Support, 7. Associations, 8. Repetition and Substitution, 10. Reward and Threat, 11. Comparison of Outcomes and 12. Antecedents. As well as each specific strategy acting as a BCT, the process of design also worked as a BCT. These included 1·2 Problem Solving; tailoring strategies to mothers’ own barriers and facilitators and planning for ways to overcome pre-empted challenges and 3·3, Social Support (emotional) and 3·2, Social Support (Practical), where the researcher supported the mothers development and ongoing implementation of such strategies^(38)^.

**Table 3:** Co-designed strategies and their corresponding behaviour change techniques

Strategy themes	Specific strategies identified by participants	Behaviour change techniques (BCT)^*^	Illustrative quote
Capability			
Providing alternatives to screens	Conversation starters Listening to the radio ‘Make believe’ restaurant experience Swapping handheld screen for toy or finger food Games that promote sitting at table (Jenga)	8·2. Behaviour substitution 12·4. Distraction	‘They like…[the game] Jenga…So, I was just thinking that could be something you know they can eat while everyone else is having their turn and then they can lean in and have their turn and keep back eating.’
Clear expectations	Discussing change before implementation (preparation) Clear, confident and consistent expectations of mealtime screen use Cold turkey and ‘tough love’ Creating new screen time routines Having a clear end goal	1·1. Goal setting (behaviour) 7·6. Satiation 8·3. Habit formation 8·4. Habit reversal	‘Once they [children] got over the idea that we [parents] weren’t giving in, they were more happy and relaxed, and were happy to give anything a go.’
Opportunity			
Contextual changes	Parents eating same meal as children Limiting snacks in front of TV before dinner Serving meals that can be shared Modelling expected behaviours	7·3. Reduce prompts/cues 13·1. Identification of self as role model	‘My husband and I need to go and sit at the table, while they’re eating as well.’
Physical environment changes	Moving meals to the table/away from TV area Handheld devices not being present at meals (out of sight, out of mind) More time outside (instead of inside where the screens are) Purchasing resources – child sized table and chairs, messy mat Reducing screen time overall (adult and child)	7·5. Remove aversive stimulus 7·3. Reduce prompts/cues 12·1. Restructuring the physical environment	‘I’m wondering if actually, moving her permanently up to the big table, because she doesn’t sit at the big table. Like she’ll just sit there for breakfast but not usually dinners and stuff. I’m wondering if that specialness of sitting at the big table might be what she needs?’
Family support	Sharing the load of mealtimes (checklist of jobs, delegating jobs) Enlisting support from family (partner or extended family) Getting kids involved in meal preparation	3·2. Social support (practical) 3·3. Social support (emotional) 12·2. Restructuring the social environment	‘Prior to this we would not…But now my husband said “no, everyone has to take their own plates to the kitchen”….They need to be independent…and whatever food we have dropped immediately, you have pick up and put on your plate.’
Motivation			
incentives	Rewards bribery	10·1. Material incentive 10·2. Material reward	‘Normally, they’ll have breakfast and if they’re having their breakfast, they can watch it until eight o’clock in the morning…But yeah, they could go on the phone or iPad a little bit maybe if they’ve got everything ready and done.’
New mealtime perspectives	Intentional patience, softness or empathy at mealtimes Having fun without screens Prioritising and protecting screen-free family mealtimes	11·2. Reduce negative emotions 11·3. Conserving mental resources	‘I think I would have to just be a bit easier on myself if, if it’s just me at home that day, I’m caught up with feeding [my baby]…If I have to have the TV on at lunchtime, then that’s okay. As long as I’m putting effort into no screens at lunch on other days, where it’s a bit easier.
Flexibility	Mealtimes: need to be flexible/need a backup plan Using screen as a last resort Starting with small changes and building up	1·1. Goal setting (behaviour) 1·4 Action planning 11·3 Conserving mental resources	‘The place to get to is no screens, but I’m not sure if there’s like a transitional period where we’re not having screens at breakfast or not having screens at dinner? Maybe, starting with dinner might be good…’

* Numbered behaviour change techniques denote their alignment with Michie and colleagues’ Behaviour Change Taxonomy (v1)^(38)^.

The most widely engaged strategies were exhibiting clear, consistent and firm expectations of mealtime screen use, modelling expected behaviours and altering the physical environment to help facilitate screen-free meals. Physical environmental change often aligned with screen type. For example, *moving meal location* to a dining table, kitchen bench or outside was engaged for many families aiming to reduce television use. In comparison, *moving devices* away from meals areas was often engaged for those families aiming to reduce handheld device use. Families with higher mealtime screen use tended to start with smaller goals, such as removing screens from one meal per pay and gradually increasing, while others who engaged in fewer screens were more likely to go ‘cold turkey’ and remove all screens at once.

### Follow up interviews: *Mothers’ experiences and reflections around reducing mealtime screen use*

All fourteen mothers completed the second interview with thirteen implementing at least one of their co-designed strategies three times or more, over the 3–4 week trial period. All mothers discussed their reflections after the trial period. Five main themes are identified in Table 4.

**Table 4:** Reflections and experiences of seeking to reduce mealtime screen use

Theme	Example quote
Consistency and parenting self-efficacy	
Low consistency and parenting self-efficacy resulted in little or no reduction in mealtime screen use	‘…going into it going, like, “Oh, I just can’t do it today. I can’t do it today” and then I just kept doing that and then got to just the point after about a week of doing that I’d be, like, “Oh, I just actually can’t take this on right now. I can’t do this right now. I’m just too tired and too stressed out”.’
Building consistency and parenting self-efficacy resulted positive change (even after a period of difficulty)	‘First off, it started off very badly, screaming and yelling. Horrible! But then it went really good and well…Probably a week after, I try and breathe and be calm, just saying, “If you want something, you have to help out.”’
Changes to foods served or consumed	
Improved (child eats more food, child more willing to try new foods)	‘Probably…less distracted and ate more of his food and he enjoyed it, which is nice for me to see.’
Declined (child eats less, increased use of easy prepare meals)	‘I find this one, she always eats better when she’s distracted or, yeah, sometimes the screen works better to get her to eat.’
Experience exceeding expectations	
Larger than expected benefits	I was surprised, I was actually really surprised, so I’m really glad I took part in it. I really got a lot out of it. It was just such a little thing, big impact, and I would have said “*No, it’s not really a problem in our house, we don’t have the TV on at mealtime*”.’
It was easier to change than expected (creating new habits, saying no, responding to child demands)	‘Honestly, for me it was just - in my mind I think it was going to be harder than it actually was. So it was, yeah, I just had to give it a go and went, “Oh, okay, we can do this”.’
Less perceived reliance on screens (we do not need them like I thought we did)	‘…you can do mealtimes with kids and not completely stress out. We’ve never had a mealtime that hasn’t been stressful. But the last couple of weeks it’s been fairly good. They’ve still got days because they’re kids, but for the most part, like I’m actually not dreading mealtimes now.
Increased value of mealtimes	
Mealtimes are more enjoyable and relaxed without screens	‘It made it feel more pleasant for me, to slow down and actually stop and sit with him, instead of trying to use that time, okay now I’ve got 10 minutes while he’s busy to try and do something else. So, it was nice to have that connection with him, it made it all the more pleasant.’
More presence and communication as a family	‘I thought that…we were communicating quite well even with the phone there, but it’s obviously such a distraction and temptation…I was surprised, the kids have really opened up a fair bit more to me, which has been really interesting to see and, I have definitely noticed a difference. I guess I’m more present, I’m not distracted.’
Provides opportunities (learning and connection)	‘…as she gets bigger, she’s not going to want to play with mum all day. So, it’s going to be our touch point during the day, I think, and I want to keep it to be that.’
Shifting established habits	
Effort required to shift habits (especially initially)	‘I learnt that it’s easier when I’m organised and prepared and so it’s not rushed…When I’m organised with the dinner and whatnot it’s a whole lot smoother than when I’m rushing around at 4:30pm trying to sort something out and then he’s mucking about so I put the telly on for him to keep him occupied. So, it’s much easier yeah when everything is organised.’
Cultural screen acceptance and abundance	‘…I’m definitely addicted to my phone…By sharing mealtimes with him more often, I’ve found other times during the day, whether it’s when I’m on the toilet or when he’s playing. I’ll grab five minutes just to have a scroll.’

#### Theme one: Consistency and parenting self-efficacy

Consistency and parenting self-efficacy were identified as underpinning mothers’ capacity to enact their goals. This was expressed explicitly, such as mothers reporting that consistently engaging in screen-free meals and pushing through difficult situations without screens was vital for change. It was also expressed implicitly, such as mothers who reported sporadic changes to mealtime screen use tending to report screen-free meals as more difficult for families to adjust to, compared with those who had more consistency. Most mothers reported that they built their consistency and self-efficacy throughout the trial period.

#### Theme two: Changes to foods served or consumed

Changes to foods served or consumed were experienced near opposing ends of a spectrum of consistency and self-efficacy. For one mother, the quality of foods served declined at screen-free meals. She anticipated high child resistance to change, a reliance on screens to distract her child while she engaged in preparing meals and limited resilience towards removing screens in time for meals. In response, she chose easier to prepare meals which she knew were liked by her children, resulting in higher consumption of discretionary, energy dense foods during screen-free meals. Mothers who expressed high levels of parenting self-efficacy and consistency reported that children were more likely to try new foods or consume a larger variety of the foods served.

#### Theme three: experience exceeding expectations

Prior to the trial phase, mothers had discussed that they expected change to be difficult. Although for many, aspects of reducing mealtime screen use brought challenges, most mothers reported change to be easier than expected. In addition, the benefits anticipated by reducing mealtime screen use were exceeded by the value mothers reported to have gained from this experience.

#### Theme four: increased value of mealtimes

Mothers who reported they were building consistency and self-efficacy perceived an increased value of mealtimes. In addition, mothers who expressed high consistency and self-efficacy tended to perceive reducing mealtime screen use to be easier and more beneficial than anticipated, which often exceeded their expectations.

#### Theme five: shifting established habits

Mothers felt their child/ren had established behaviours which were difficult to change and shifting established habits took considerable effort, particularly initially. This including being more organised, more present and more engaged with children. Those reporting high consistency and resilience worked through this stage more easily. Many mothers acknowledged the importance of reducing their family’s mealtime screen use now, while their child was still young and had spent less time building habitual mealtime screen use. Those with less ingrained habits also appeared to find change easier. For example, for the two families in this study who reported not having access to a dining table, the family whose limited access had been a long-term scenario, (i.e. had been eating meals in the living room for some time), found it difficult to move mealtime location. In comparison, the family who had more recently lost access to their dining table, because of COVID-19 related remote working/learning arrangements, found moving mealtimes back to the dining table a feasible option.

Families with older children found value in engaging them for social support. Mothers reported, in part, cultural screen acceptance and abundance heavily enabled mealtime screen use. This included a learned reliance on and addiction to screen use more broadly, both in their children and themselves (for example, parent mobile phone addiction and the difficulty of ignoring phone notifications). One highly motivated single mother participated in MI and co-designed several strategies but did not participate in the trial phase of the study. However, in the follow-up interview she reported ‘I was very gung-ho on the day and my enthusiasm waned very quickly’. She reflected on her perceived low resilience and consistency, including self-reported limited capacity to cope with current life stressors (mental health and relationship challenges), family stability and mealtime routine, which limited her capacity to overcome barriers to screen-free meals.

#### Recommended supports and future plans

Participants were asked about resources that may have supported them to make change. Suggestions included reminders, mealtime planning tools, tips for involving children in meal preparation, managing child resistance and ways to redirect children when they ask for screens, as well as the usefulness of having partners involved in the co-design phase. All thirteen mothers who implemented strategies had plans to continue. Some reported a previous desire for reducing their mealtime screen use before beginning the study but had been unsure how to implement change.

## Discussion

Using a combination of MI and co-design techniques, this qualitative study explored mothers’ perceptions and experiences of reducing mealtime screen use in families with young children, in a low SEP sample. Mothers co-designed a large number of feasible strategies in line with established BCT, tailored to the challenges of family meals and aimed at reducing mealtime screen use. Mothers’ experiences were driven by their level of consistency and self-efficacy to change and included changes to foods served or consumed and increases in the value of mealtimes. Overall, mothers found that beneficial changes were feasible and easier to enact than anticipated. Regardless, shifting established habits did require considerable effort. Almost all mothers reported successes in increasing the number of screen-free family meals and planned to maintain changes within their home, regardless of other demographic factors.

Consistent with a realist synthesis of obesity-related behaviour change interventions^(42)^, this study identified three specific strategies frequently used by families: increasing opportunity through altering the physical environment; role modelling healthy mealtime behaviours and exhibiting clear, consistent and firm expectations of mealtime screen use. While there was commonality among approaches taken, it was also the case that many additional BCT were used across the group. This is likely to reflect the differing challenges families seek to address and highlights the potential benefit of developing tailored intervention strategies.

One such strategy, altering the physical environment by consistently having any meals or snacks at a dining table or bench space (usually moving them from a TV or lounge area) was reported to be particularly helpful for families in reducing mealtime TV use. Previous research has identified cross-sectional associations between eating at a table and better dietary and psychosocial outcomes in children^(43,44)^. Likewise, emerging prospective evidence suggested that the relationship between children’s mealtime TV use and later intakes of discretionary foods differed by mealtime location^(18)^. Families were also able to utilise meal location to set screen use boundaries and implement new mealtime expectations, such as ‘no screens at the table’. This then also offered increased opportunity through modelling. Parental modelling of expected behaviours, such as joining in on screen-free meals, was a useful strategy caregivers utilised in this study and is a well-established strategy for encouraging child behaviours^(8)^. These findings highlight the potential for nutrition promotion initiatives to encompass location of eating in nutrition messaging. However, findings also suggest that screen-free family meals might be more difficult to achieve for those with limited opportunity to alter their physical environment. Messaging should consider socio-economic implications, such as owning or having space for a dining table, so that healthy mealtimes feel achievable, regardless of family resources.

Consistent with utilising changes to the physical environment and parental modelling as potential BCT, reducing parent phone use at family meals was a key goal for many families in this study. Parental phone use at mealtimes may contribute to normalising mealtime screen use behaviours as well as distracting parents from engaging with their child. One recent survey of 298 parents of toddlers living in Norway found 40 % of parents reported using their phone at mealtimes which was associated with unhelpful feeding practices, such as applying pressure to eat, and reduced likelihood of engaging in family meals^(45)^. These findings and those in the current study highlight a need for increasing caregiver awareness around the importance of screen-free meals, including limiting phones during mealtimes.

This study suggests that a reduction in use of screens at mealtimes may have nutrition trade-offs for some, which requires exploration. Screens may not only be used for behaviour management^(20)^ but also to facilitate the consumption of more nutrient dense foods^(46)^. Nonetheless, the current study suggests that some of the more difficult aspects of reducing mealtime screen use, such as the increased effort required to shift established habits, might be improved with consistent screen-free meals and increased caregiver confidence. These changes could offer potential pathways to work with these families to produce more beneficial outcomes. Longer-term trials are required to determine if reducing mealtime screen use can be sustained and the nutritional impacts of such change.

Interventions aimed at reducing family mealtime screen use in families with young children could take advantage of mothers’ high value of family meals for providing opportunities for communication, family connection and learning opportunities. In addition, even though mothers in this study were likely highly motivated to reduce their mealtime screen use, many reported that it took significant effort, especially initially. Most mothers discussed that normalising screen-free behaviours by avoiding the development of mealtime screen use habits altogether was likely to be easier than trying to reduce them later in life, which is supported by past literature^(43)^. These findings emphasise the need for promoting screen-free meals from the inception of mealtimes, opportunities for education and the design of targeted strategies that support the adoption of screen-free meals.

This is the first study to explore caregiver perceptions of reducing mealtime screen use in families with young children. Focusing on families of lower SEP has offered insight into how we might engage families from diverse backgrounds in initiatives aimed at limiting mealtime screen use. A key strength of this study was the use of MI and co-design methods. This process enabled participants to explore their own motivations and commitment to change^(36)^ and co-construct ideas based upon the participants’ lived experiences and the researchers’ scientific knowledge^(28)^, strengthening engagement and ownership of the behaviour change process. Although it is likely some self-selection bias played a role in mothers’ pre-trial motivation levels, it appeared that MI helped to consolidate motivation by encouraging mother’s to self-identify their capability and opportunity for change.

Despite all participants being mothers and the acknowledgement that fathers are an important but missing inclusion in this research^(47)^, this study was successful in recruiting a diverse sample of motivated participants. In Australia, mothers are more often responsible for the preparation and management of meals^(48)^. Therefore, results of this study are likely transferable to other similar family groups and highlight the potential, not only to engage culturally and socioeconomically diverse mothers in behaviour change, but in the promotion of screen-free meals. Notably, most mothers had completed a trade or diploma. Therefore, education levels were on the higher end of ‘low SEP’, within the approximately 50 % of women aged 25–34 years in Australia without a university education^(49)^. This may limit some transferability to groups whose highest completed education levels include high school or lower. Further research could explore families with lower educational background and, where appropriate, include both caregivers’ in the process of behaviour change planning. It is also important to note that although efforts were made to confirm the accuracy of researcher interpretations with participants, recounts of participant experiences are acknowledged to be influenced by the role of the researchers in this study.

A final consideration is the timing of data collection. Given that this research was conducted during the COVID-19 pandemic, some participants were experiencing higher than usual amounts of stress and screen time. During the COVID-19 pandemic, overall screen use in Australia increased,^(50)^ and it is possible that mothers considered screen related behaviours temporary as their family adjusted to living under lockdowns. While this may not be reflective of usual, it also shows that mothers had capacity to engage in such behaviour change, even during a challenging time.

Findings from this study could be used to inform future interventions aimed at using the family meal as a setting to promote optimal nutrition to children and families, providing acceptable suggestions to limit mealtime screen use and optimise opportunities for healthy outcomes. Embedding the promotion of screen-free meals into existing early childhood nutrition promotion initiatives may be beneficial. Many strategies were used consistently across families, regardless of the type of screens being used. For example, strategies such as parental modelling of screen-free meals, sitting to eat together (at a table, bench or mat on the floor) and getting the family invested and helping at mealtimes. These strategies may be well received by a majority of mothers and help motivate families to engage in healthy family meals. This requires testing on a larger scale.

At an individual level, this study highlights the value of early life practitioners understanding family-level barriers, to assist in making feasible recommendations around the promotion of healthy family mealtimes. While time-intensive MI sessions are unlikely feasible, their intent in this study was the generation of co-designed strategies for future testing which, at a broader scale, may offer a suite of realistic strategies practitioners could promote as part of routine, person-centred care. Furthermore, there is a need for developing and testing accessible and realistic resources for caregivers and families around ways to limit mealtime screen use.

## Conclusions

This qualitative exploration of mothers’ perceptions and design of strategies around reducing mealtime screen use in families with young children has highlighted a number of feasible strategies caregivers might engage. Several strategies appear likely to be transferrable, including moving meals away from TV areas to a table or bench, having consistent expectations at mealtimes and promoting healthy mealtime behaviours through parental modelling of screen-free meals. Small, incremental changes appeared acceptable for families with high mealtime screen use. These strategies should be tested for their effectiveness in a larger sample. Finally, although reducing family mealtime screen use appeared feasible in these motivated families, it took considerable effort to implement change. This challenge highlights the importance of pre-emptive messaging and embedding the promotion of screen-free mealtimes into nutrition promotion from early in a child’s life.

## Supporting information

Litterbach et al. supplementary material 1 Litterbach et al. supplementary material

Litterbach et al. supplementary material 2 Litterbach et al. supplementary material

## Supplementary material

For supplementary material accompanying this paper visit https://doi.org/10.1017/S1368980023002847
