# Supplementary material for: Mothers’ experiences of reducing family mealtime screen use in Australian households with young children
Source: Public Health Nutr. 2023 Dec 15;27(1):e21. doi: 10.1017/S1368980023002847 (PMC10830358; doi:10.1017/S1368980023002847)
Supplement: Litterbach et al. supplementary material 1 — Litterbach et al. supplementary material [file S1368980023002847sup001.docx]

## Additional file 1. Relevant interview questions

*(Consent and study background text removed)*

****

I wanted to mention that partway through the interview, I will ask you about whether or not you would like to be part of further research in this area and there is absolutely no obligation to do so.

*****

Is reducing screen time during family mealtimes something you and your family might be interested in doing? ****If no, move to different set of questions (not reported in this paper)*

(if yes) If there was anything about your mealtime screen use you wanted to change, what would it be?

Can you think of any strategies that you think might help in trying to limit Mealtime screen use/[behaviour mentioned above]?

What do you think will be hard about doing this?

What do you hope would change if you reduced screens at mealtimes?

What do you think would actually happen if you removed screens at mealtimes?

*****

Thank you very much for your time and input around your views on mealtimes and screen use, today. For being part of this interview, you will receive a $40 Woolworths voucher.

Before you go, I’d like to offer you the opportunity to work with me to develop some strategies for reducing mealtime screen use in your own home, and with my support, trial these in your home? I’d like to reassure you that there is no obligation to take part in this, if you do not wish to.

Would you like to hear about this? ****If no, go to other questions*

We would like to know if you would be able to work with me to develop some strategies to trial over a 2-3 week period, to see how useful and appropriate they are for reducing mealtime screen use.

Then we would like to check back in for a discussion, in a few weeks’ time, to see how you went. Participants will receive an additional $40 Woolworths voucher for completing this part of the study. You can trial the strategies as many or as few times as you like.

Do you think you would be interested in doing that?

****If no, go to other questions*

Thank you very much. Let’s quickly recap the strategies you suggested earlier.

******OR if they didn’t suggest any strategies***

1. Can you think of any strategies which you could introduce into your mealtimes to help reduce the amount of screens your family uses?

******OR if they can’t think of any strategies***

1. There are a few strategies I could suggest that have been used by other families and you could let me know if you think any of these might work in your family?
   1. Start by moving the meal into a room without the TV (could make it special/exciting, a picnic on the floor or bring a Teddy bear to dinner?
   2. Set a timer to see if the family can eat without screens until the timer goes off

**** Here we will provide suggestions on child feeding practices where relevant in line with Satter’s Division of Feeding Responsibility model (1).*

****We will then spend a few minutes going over the strategies put forth and plan them out a little more.*

Prompt: How can we address the barriers to screen free meals which we discussed earlier?

Prompt: Let’s set a couple of goals around limiting screens during meals

- For example, I would like to have 3 breakfasts per week without TV?

Reminder: new things won’t necessarily work the first time you try them, especially for children who like a familiar routine. Try not to feel disheartened! You might have to try something a few times to make a new routine.

While you are trialling these strategies, try to occasionally jot down how things are going, what’s working well and what isn’t working. And if you think of or come up with any ideas about resources, such as posters, reminders, etc., that might help you, jot them down too, so we can discuss them when we chat again. You could write them on a scrap of paper and stick them on the fridge or put a note in your phone?

I will send you an email after we complete this interview recapping the strategies we discussed and the goals you set. I will include the $40 Woolworths gift card as a thank you for being part of this interview. And I will send you the second gift card after the second, shorter interview.
