# Supplementary material for: Mothers’ experiences of reducing family mealtime screen use in Australian households with young children
Source: Public Health Nutr. 2023 Dec 15;27(1):e21. doi: 10.1017/S1368980023002847 (PMC10830358; doi:10.1017/S1368980023002847)
Supplement: Litterbach et al. supplementary material 2 — Litterbach et al. supplementary material [file S1368980023002847sup002.docx]

## Additional file 2. Follow up semi-structured Interview schedule

*(Consent and study background text removed)*

1. Let’s do a quick recap of the strategies we talked about in our last interview…
2. How did you go with the strategies we talked about?
   1. What worked well?
   2. What didn’t work well?
   3. What was the biggest challenge?

| Capability | 1. What did other family members think about the strategies?    1. Children    2. Partner 2. What did you learn (if anything) about mealtimes in your home during the last few weeks? |
| --- | --- |
| Opportunity | 1. If you used specific strategies to help you (excluding the tips sheet), how (if at all) did the strategies support you in reducing your mealtime screen use?    1. For example, if you used a timer or reminder system, was this helpful? Why/why not?    2. For example, if you wanted to start meals without the TV, how hard was it to do so, did you turn it on again during your meal/ after your goal time? 2. If you used the tips sheet, how (if at all) did it support you in reducing your mealtime screen use? |
| Motivation | 1. How (if at all) did implementing these strategies change the way you feel about mealtimes?    1. Mood ?    2. Enjoyment ? |
| Behaviour | 1. In what ways (if any) did mealtimes change? 2. In way ways (if at all) did mealtime screen use change as a result of trying these strategies? 3. In what ways, if at all, did changing your screen use during mealtimes, change the foods that you ate during mealtimes?  (Middleton et al. 2020 systematic review) 4. How likely is it that you will continue with these strategies? |
| Capability | 1. Can you think of anything else that would have been helpful for you to have in your home to help implement the strategies you trialled?   For example,   1. a list of conversation starters 2. Games to play around the table 3. Tips on getting the kids involved with preparation/clean up |

1. Is there anything that you observed during mealtime over the last few weeks that we haven’t discussed today?
2. Is there anything else you would like to add about family meals or mealtime screen use generally?
